# Salvaging Pull-Out Strength in a Previously Stripped Screw Site: A Comparison of Three Rescue Techniques

**DOI:** 10.3390/jfmk6030071

**Published:** 2021-08-27

**Authors:** Francesco Addevico, Giovanni F. Solitro, Massimo Max Morandi

**Affiliations:** 1 Department of Orthopedic and Traumatology, Niguarda Hospital, 20162 Milano, Italy; 2 Department of Orthopaedic Surgery, Louisiana State University Health-Shreveport, Shreveport, LA 71103, USA; massimo.max.morandi@gmail.com (G.F.S.); maxmorandi@aol.com (M.M.M.)

**Keywords:** screw, stripping, rescue, thread

## Abstract

Screw stripping during bone fixation is a common occurrence during operations that results in decreased holding capacity and bone healing. We aimed to evaluate the rescue of the stripped screw site using screws of different dimensions. Five screw configurations were tested on cadaveric specimens for pull-out strength (POS). The configurations included a control screw tightened without stripping, a configuration voluntarily stripped and left in place, and three more configurations in which the stripped screws were replaced by a different screw with either increased overall length, diameter, or thread length. Each configuration was tested five times, with each screw tested once. The POS of the control screw, measured to be 153.6 ± 27 N, was higher than the POS measured after stripping and leaving the screw in place (57.1 ± 18 N, *p* = 0.001). The replacement of the stripped screw resulted in a POS of 158.4 ± 64 N for the screw of larger diameter, while the screws of the same diameter but increased length or those with extended thread length yielded POS values of 138.4 ± 42 and 185.7 ± 48 N, respectively. Screw stripping is a frequent intraoperative complication that, according to our findings, cannot be addressed by leaving the screw in place. The holding capacity of a stripped screw implanted in cancellous bone can successfully be restored with a different screw of either larger diameter, longer length, or extended thread length.

## 1. Introduction

A common problem encountered in orthopaedic surgical procedures is screw stripping due to overtightening, because a stripped screw offers poor fixation [1]. Fletcher et al. reported 26% of screws inserted by surgeons in various bones were irreparably damaged due to overtightening, leading to the failure of the screw-bone interface [1]. Furthermore, a study by Stoesz et al. reported rates of screw stripping performed by surgeons in cancellous bone to be as high as 45.4% [2]. Studies have been conducted on how surgeons tighten screws based on “subjective feel”, highlighting the commonness of this human error [3].

The existing literature offers several strategies that can be employed in the case of screw stripping, such as plating [4], augmentations with screws [5] or bone void fillers [6]. However, all of these strategies described are more invasive, and there are anatomical areas for which these options are not convenient or feasible, such as the sacroiliac joint [7] and femoral neck, where specific screw configurations for optimum stability are required [8]. Few authors have previously tested the option of saving the original site of the stripped screw, and these few existing studies have yielded controversial results regarding successful rescue strategies [9]. The majority of these studies are focused on the stripping of cortical screws rescued with larger cancellous screws [9,10]. One study attempted augmenting the screw site with suture wire but could not prove that such augmentation provided clinically significant improvement over the original screw’s pull-out strength (POS) [10]. Bone cement is widely used for surgical procedures involving cancellous bone [11]. However a limit of this technique is the inability to control cement migration to unwanted locations, thus creating potential for neurological damage [6]. Though rescue of cortical bone screws has been widely addressed, a comprehensive evaluation of less invasive rescue strategies for cancellous bone has not yet been proposed but is needed, such as in the case of femoral neck, sacral, or tibial fractures that are often threaded with screws relying on their capacities of compression [6].

With the aim of exploring rescue approaches for cancellous bone that are less invasive than bone cement, in this study, we compare the holding capacity of the primary screw with three rescue strategies that do not require alternative holes or supplemental fixation. We hypothesized that despite the bone damage due to stripping, pull-out strength can be recovered in the same location of the stripped screw using a screw of the same type, but of different dimensions.

## 2. Materials and Methods

The study was performed according to the rules of the institution ethical committee.

### 2.1. Study Design

In this experiment, the screw pull-out strength (POS) was used to judge fixation quality [12] and strength (POS) of five configurations. The screw implanted without stripping acted as the *control* configuration. A second configuration was constituted by the screw voluntarily *stripped* at time of insertion and left in place. The three rescue configurations were recreated by intentionally stripping the screws and replacing them with screws of either *larger* diameter, *longer* length, or with an *extended thread*. All tests were performed on human cadaveric specimens harvested from donors with mean age of 65.2 ± 8 years old. The proximal tibial metaphysis of 25 human cadaveric specimens were used as models of human cancellous bone [13]. CT scans were performed using a GE LightSpeed VCT at 120 kV, 100 mA and 1.25 mm prior to testing in order to assess specimen absence of previously implanted hardware. The distal part of the tibiae was cemented using polyester resin (Bondo, 3M, Maplewood, MN, USA) in a metallic box to constrain the specimens during testing. These were then randomized to the five test configurations. Each configuration was tested five times with each specimen and each screw tested once.

### 2.2. Screw Characteristics

**Figure 1:**
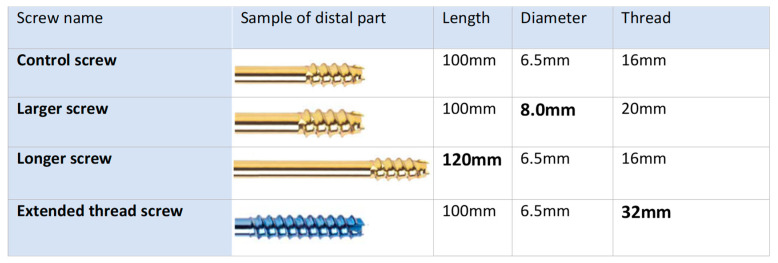
Characteristics of the screws considered in the current study.

Titanium cannulated screws (Rondò, Citieffe; Bologna, Italy) were chosen with dimensions associated with each configuration (see Figure 1). The control screw was 100 mm in length and 6.5 mm in diameter with a thread length of 16 mm from the tip and constant pitch of 2.75 mm. The diameter of this screw was chosen in order to increase surgeon perception of tightness as suggested in existing literature [14]. From this reference dimension, the three rescue configurations had the following characteristics: the *larger screw* had the same length and thread as the control screw but a diameter of 8 mm, the *longer screw* had the same diameter and thread of the control screw but a length of 120 mm, and the *extended thread screw* had same length and diameter of the control screw but a thread extension of 32 mm.

### 2.3. Screw Insertion

**Figure 2:**
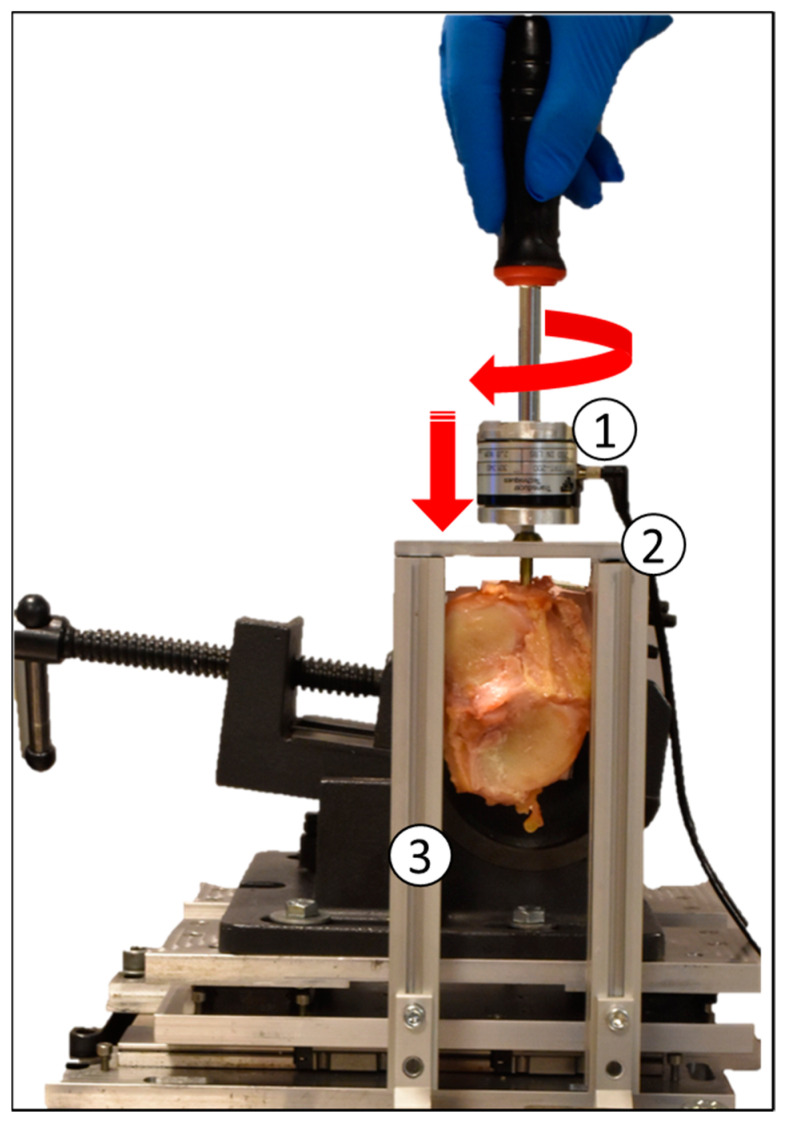
Experimental setup used for the screws insertions that includes a torque meter sensor (1), a spacer to ensure screw placement consistency (2), and an adjustable support for positioning (3).

An experienced orthopaedic surgeon implanted each of the screws by hand 10 mm below the articular surface of the tibia with a gap of at least 5 mm from the medial cortex. Each screw was tightened in correspondence of the tibial coronal plane along the lateral to medial direction. An adjustable spacer was used to ensure consistency in screw placement (see Figure 2). Following the surgical technique, each hole was pre-drilled by a 3.5 mm cannulated hand drill with a 1.8 mm K-wire as a guide. The screwdriver was instrumented with a torque meter (TRT-200, Transducer Techniques, Temecula, CA, USA) that measured the torque over time.

**Figure 3:**
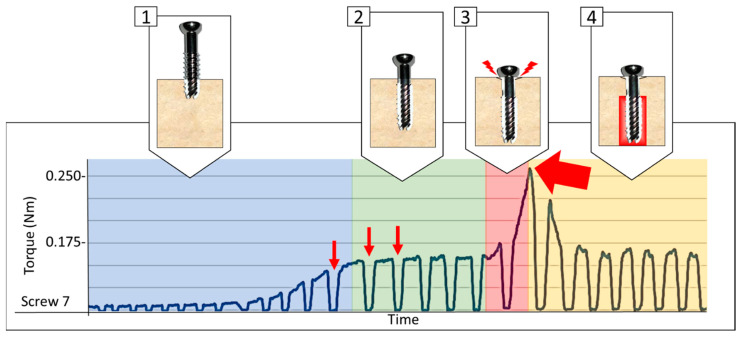
Example of torque [Nm] measured during instrumentation of the screws depicted in phases: (1) Insertion of the thread; (2) Screw advancement at near constant torque; (3) Compression phase: engagement of the cortex reaching maximum torque; and (4), Torque decreased to near constant value following the stripping.

The *control* configuration constituted by the screw inserted without stripping was obtained by screwing up to about 50% of the peak torque that was observed in the beginning of the compression phase (see Figure 3) [15].

### 2.4. Screw Stripping and Rescue Techniques

The four remaining configurations were obtained by tightening a first screw past the compression phase (phase 4 of Figure 3) until a drop from the torque peak value was observed [16,17,18,19]. 

The three rescue configurations were conducted by unscrewing and removing the stripped screws, then repeating the insertion procedure with the three rescue screws. With the K-wire placed again as a guide, the rescue screw was implanted using the same entry.

### 2.5. Mechanical Testing

**Figure 4:**
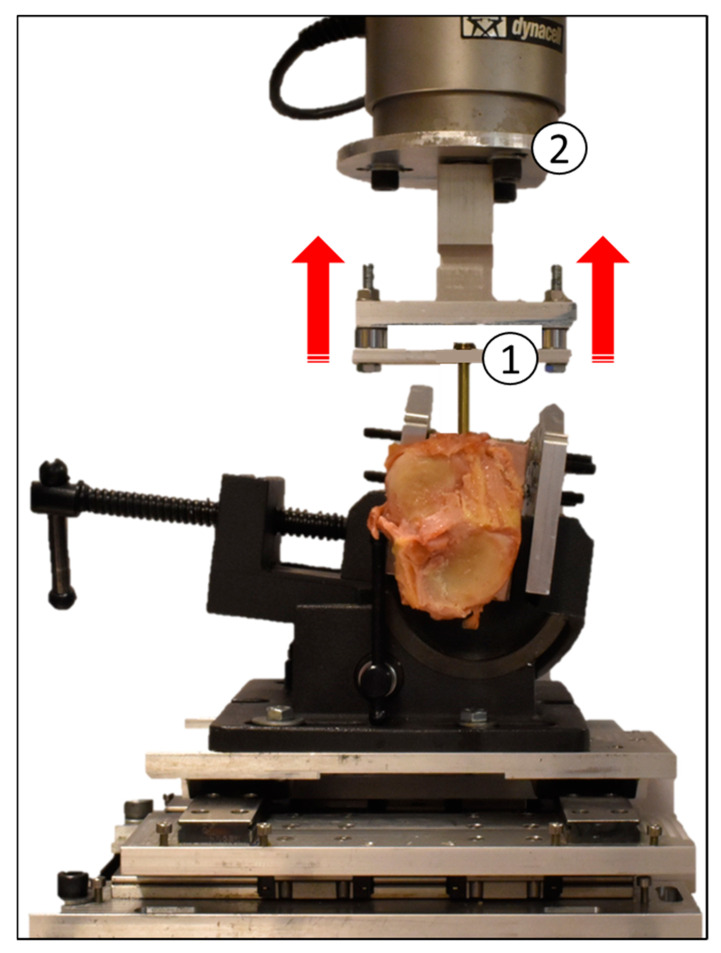
Mechanical testing for screw pull-out strength (POS), in which the screw is pulled through the custom-made fixture (1) and attached to the load cell (2) while the bone, supported by a system of rails, is free to move in the X-Y plane.

Following insertion, each screw was tested for pull-out strength by imposing an axial displacement at a rate of 5 mm/minute through a universal testing machine (Instron E3000, Norwood, MA, USA) in accordance with the Standard Specification and Test Methods for Metallic Medical Bone Screws (ASTM F543). The peak force values recorded during the extraction provided the POS of the screws (see Figure 4).

### 2.6. Statistical Analysis

Differences among configurations were analysed with ANOVA, while specific differences between groups were identified using Student’s *t*-test. The significance level of all the analyses was set at *p* < 0.05 (two-tailed). All statistics, descriptive and analytic, were performed using SPSS (v24.0, Armonk, NY, USA).

## 3. Results

**Figure 5:**
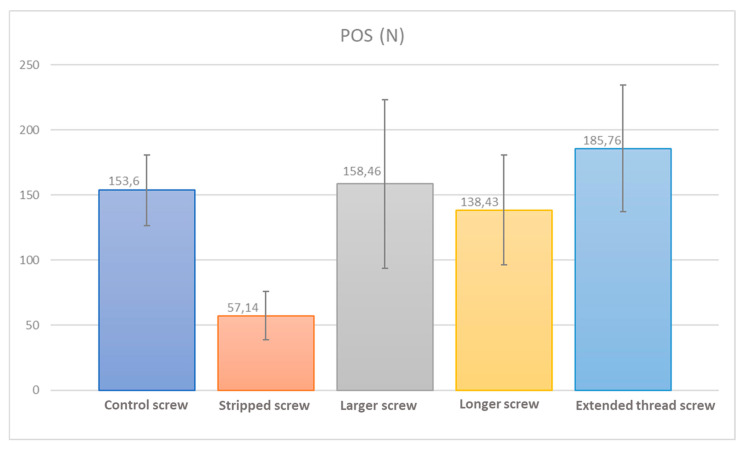
Experimentally obtained pull-out strength values for the considered configurations.

CT analyses did not yield bony defects or hardware in any of the specimens. The mean attenuation coefficient was 206.3 ± 48 HU (Hounsfield Units) and no differences in density were reported among groups (*p* > 0.05). None of the 25 used screws were damaged during testing. The *stripped screws* resulted in a peak torque of 0.74 ± 0.3 Nm, which was higher than the *control screw* torque of 0.36 ± 0.1 Nm (*p =* 0.018). The rescue screws were inserted with a torque of 0.31 ± 0.01 Nm, 0.19 ± 0.1 Nm and 0.24 ± 0.1 Nm, respectively, for the *larger screw*, *longer screw*, and *extended thread screw*. The POS of the control screw, measured to be 153.6 ± 27 N, was higher than the POS measured after stripping and leaving the screw in place (57.1 ± 18 N, *p* = 0.001). All three rescue strategies were able to recover the POS to the level of the control (*p* > 0.05). The *extended thread screw* yielded the highest POS (185.7 ± 48 N, *p* = 0.002), while the POS for the longer screw and larger screw were limited to values of 158.4 ± 64 N (*p* = 0.011) and 138.4 ± 42 N (*p* = 0.016), respectively (see Figure 5). No statistically significant differences in the POS of the rescue screws were found (*p* > 0.05).

## 4. Discussion

Poor stability and low compression in the site of fracture have been related to a higher rate of late consolidation, non-union, and chronic pain in multiple scenarios [20,21]. Sufficient compression is important to achieve optimal stabilization of the fracture, and lag screws allow for maximum compression on the direct surface of the bone [22]. Pull-out strength (POS) directly correlates with screw holding strength, which increases with compression. Thus, POS can be used as a measurement of compression [23]. We found that the POS for the 6.5 mm screw implanted in cancellous bone decreased from the value of 153.6 ± 27 N for the intact screw to 57.1 ± 18 N (62.8 ± 12%, *p* = 0.001) for the stripped screw. Comparably, Collinge et al. and Marmor et al. reported a strength reduction ranging from 76% to 82% when stripping occurred while testing screws of 3.5 mm and 4 mm in a bone substitute [9,24]. These results suggest that leaving the stripped screw in place should be not considered as a valid option during orthopaedic surgical procedures.

The main finding of this study is that the holding capacity of a stripped screw in cancellous bone can be restored by replacing it with a screw either longer in length, larger in diameter, or with an extended thread, thereby salvaging the original screw site. The larger implanted screws replacing the stripped screws had values of POS of 158.4 ± 64 N. Those of same diameter but longer or those with extended thread yielded POS values of 138.4 ± 42 and 185.7 ± 48 N, respectively. In general, our findings show that having more depth available to the stripped screw would allow the implantation of a longer screw, while a larger segment under fixation would allow the use of a screw larger in diameter. The cannulated screw type used in our study can be used in femoral neck fractures, which have a similar composition to the proximal tibia in that both are composed of cancellous tissue [25]. Considering the specificity of each anatomical district of orthopaedic interest, further site-specific investigations are needed to interpret our results for particular clinical applications. Lag screws ranging between 6 mm and 8 mm of diameter are the most commonly adopted in many orthopaedic surgical procedures, including sacroiliac trauma fixation [7,26], femur neck fixation, and tibial plateau fractures [8,27]. The present study was performed on the proximal epiphysis of the tibia because Fletcher et al. indicated a need for screw stripping studies performed on cancellous bone due to its lesser density as compared to cortical bone, a characteristic which contributes to the risk of overtightening [1]. We therefore selected the proximal epiphysis of the tibia due to its high content of cancellous bone [28], making it an appropriate model of human cancellous bone [13]. Therefore, our results can be generalized to any threaded screw connection performed within cancellous bone, since variability in bone density among epiphyseal bones of various skeletal sites has been found to be negligible [29]. Screw stripping is a common problem encountered during operations, with several rescue strategies proposed in the literature [9,10,17,19]. A previously reported study by Collinge et al. suggested the use of a larger (4 mm) cancellous screw to replace a smaller (3.5 mm) stripped cortical screw [24]. However, the use of a larger diameter screw alone as a rescue strategy was not corroborated by the in vivo study by Wall et al., who reported that only a small fraction of the original purchase is achieved by this procedure [30]. Different from previous studies, we focused on rescuing the site of a trabecular bone screw with a screw of the same design. Another important aspect to mention is that in our study, screws were manually inserted in accordance with the manufactured surgical technique (Citieffe, Bologna, Italy). The specific influence of a power-assisted tool in determining the screw holding capacity was not evaluated in this study, because it has already been proven that no differences between manual and power-assisted tapping of cortical screws exist [31]. The clinical applicability of the findings is limited by the ability of the surgeon to recognize that stripping occurred. In a study conducted by Stoesz et. al, it was found that blindfolded surgeons were able to recognize a stripped screw in only a small amount of cases [2].

## 5. Conclusions

In conclusion, we found that screw stripping is a frequent intraoperative complication that cannot be addressed by leaving the screw in place. The holding capacity of a stripped screw implanted in cancellous bone can successfully be restored with a different screw of either larger diameter, longer length, or extended thread length. The specific choice among these three rescue screw configurations should be made by considering the morphometry of the specific anatomical site and object of instrumentation.
